# Ediacaran discs from South America: probable soft-bodied macrofossils unlock the paleogeography of the Clymene Ocean

**DOI:** 10.1038/srep30590

**Published:** 2016-07-27

**Authors:** María Julia Arrouy, Lucas V. Warren, Fernanda Quaglio, Daniel G. Poiré, Marcello Guimarães Simões, Milena Boselli Rosa, Lucía E. Gómez Peral

**Affiliations:** 1 Centro de Investigaciones Geológicas – CONICET – FCNyM (UNLP), Diagonal 113 N°275, La Plata, Argentina; 2 Departamento de Geologia Aplicada, Instituto de Geociências e Ciências Exatas, Universidade Estadual Paulista, Avenida 24A, 1515, Rio Claro 13506-900, Brazil; 3 Curso de Geologia, Instituto de Geografia, Universidade Federal de Uberlândia, Rodovia LMG 746, Km 1, Monte Carmelo 38500-000, Brazil; 4 Departamento de Zoologia, Instituto de Biociências, Universidade Estadual Paulista, Distrito de Rubião Júnior, Botucatu 18618-000, Brazil

## Abstract

The origin, affinity and paleoecology of macrofossils of soft-bodied organisms of the terminal Ediacaran Period have been highly debated. Previous discoveries in South America are restricted to small shelly metazoans of the Nama Assemblage. Here we report for the first time the occurrence of discoidal structures from the Upper Ediacaran Cerro Negro Formation, La Providencia Group, Argentina. Specimens are preserved in tabular sandstones with microbially-induced sedimentary structures. Flute marks and linear scours at the base of the sandstone layers indicate deposition under high energy, episodic flows. Stratigraphic, sedimentologic, petrographic and taphonomic analyses indicate that the origin of these structures is not related to abiotic process. Preservational and morphological features, as invagination and the presence of radial grooves, indicate that they resemble typical morphs of the *Aspidella* plexus. The large number of small-sized individuals and the wide range of size classes with skewed distribution suggest that they lived in high-density communities. The presence of *Aspidella* in the Cerro Negro Formation would represent the first reliable record of Ediacaran soft-bodied organisms in South America. It also supports the paleogeographic scenario of the Clymene Ocean, in which a shallow sea covered part of the southwest Gondwana at the end of the Ediacaran.

Macroscopic fossils ascribed to soft-bodied organisms^1^,^2^ found in terminal Neoproterozoic rocks (Ediacaran, 635–541 Ma) are among the earliest records of morphologically complex life forms^3^,^4^. These fossils may represent a mixture of stem- and crown-group metazoans, as well as extinct kingdom of eukaryotes^5^ or higher order clades with no modern representatives. They are preserved as impressions with distinct taphonomic modes or styles, and are grouped into the Avalon, White Sea and Nama assemblages^6^. Fossils assigned to the Avalon assemblage (575–560 Ma) are best known from various localities in Eastern Canada and from the Charnwood Forest, England, and may represent an early evolutionary stage of the morphologically complex macroscopic organisms. Despite their low taxonomic richness, the Avalon organisms represent forms preserved *in situ* that thrived in deep-water marine settings (but see^7^ and references therein for distinct interpretations), nearly 5 Ma after the 580 Ma Gaskiers glaciation^4^. The younger White Sea assemblage (560–550 Ma) holds the highest taxonomic diversity, with the best-preserved and most-diverse occurrences recorded in deposits of Australia (Flinders Ranges) and Europe (White Sea coast of Russia), typically in marine settings above storm wave base with seafloors covered by microbial mats^8^. The Nama assemblage, although Ediacaran (550–541 Ma), is distinct from the younger assemblages as it records the earliest known members of calcified metazoans (e.g., *Cloudina, Corumbella*) instead of soft bodied organisms and with an apparent decline in taxonomic richness^5^,^6^.

To date few dozens of species assigned to soft-bodied, macroscopic discoidal organisms ascribed to typical Ediacaran holdfast structures have been described from several localities in major continents^2^. The taxonomic and evolutionary affinities of those discs have been highly debated and assigned to microbial colonies^8^, individual^2^ and frondose organisms^9^, and even convergent styles of preservation of distinct organisms^10^. Except for rare examples in which the disc-shaped forms were found attached to their corresponding fronds, the majority of specimens reported are solely discs; the difference in number of discs and fronds has been tied to taphonomic and biologic (i.e., onthogenetic) reasons (e.g., refs 9, 10, 11, and references herein). Here we describe soft-bodied discoidal specimens from the La Providencia Group, Buenos Aires Province, Argentina, with several typical features of macrofossils of the Ediacaran *Aspidella* plexus^9^. The new finding adds information on the distribution and preservational style of Ediacaran fossils worldwide. Also, it helps to resolve the paleogeographic scenario of the southwestern Gondwana during the terminal Neoproterozoic, in which the Río de la Plata Craton reached the west coast of the Clymene Ocean as part of the southeastern Gondwana.

## The South American record of Ediacaran macrofossils

Pioneer works^12^ and subsequent advancing reports in the 80’s^13^,^14^,^15^ have revealed the presence of remains of probable biological origin in South American Neoproterozoic successions^7^,^16^. Those, however, are limited to calcified metazoan remains, trace fossils, algae, acritarchs and vendotaenids and are scarce and poorly documented when compared with typical Ediacaran assemblages recorded in Africa, Australia, Canada, England and Russia.

The most diverse South American assemblage of Neoproterozoic body fossils was described from the late Ediacaran Corumbá Group, Mato Grosso do Sul, Brazil, and includes acritarchs^1^, the calcified or organic-walled macroscopic *Cloudina lucianoi*^14^*, Corumbella werneri*^13^ and extremely rare conulariids^17^. Recent discoveries of skeletal organisms of the Nama Assemblage considerably encouraged the study on taxonomy, taphonomy and paleoecology of Ediacaran assemblages in South America^18^,^19^,^20^,^21^.

Apart of skeletal records of acritarchs^16^,^18^, calcified or organic-walled macroscopic *Cloudina lucianoi*^14^*, Corumbella werneri*^13^ and extremely rare conulariids^17^ recorded in the Ediacaran Corumbá Group, Mato Grosso do Sul, Brazil, impressions of macroscopically complex, soft-bodied organisms are scarce in South American deposits of this age^22^,^23^,^24^. Siliciclastic sedimentary strata preserved in the Neoproterozoic Itajaí Basin, southern Brazil, record poorly preserved impressions assigned to *Parvancorina* sp., *Charniodiscus*? sp. and *Cyclomedusa* sp^23^. On the other hand, supposed impressions of soft-bodied specimens were reported from a fluvio-marine succession in the probably Cambrian Jaibaras Basin, northeastern Brazil^24^. However, the nature of those “impressions” is still controversial, mainly due to: (a) their extremely poor preservation, (b) the deep weathering of structures and rock matrix, (c) their preservation within a fluvio-marine succession, (d) the low number of structures in the same bedding plane (except for the Jaibaras Basin), in clear contrast with their large abundance in the best known Ediacaran occurrences^4^, (e) their much larger sizes when compared with other well-accepted records, and (f) unreliable depositional ages of the units containing the dubious structures or fossils (especially in the case of the Jaibaras Basin). In other circumstances, such as the Santa Barbara Formation, Camaquã Basin, southern Brazil, the exclusively continental depositional environment of the successions^25^ inhibits the occurrence of fossilized marine organisms. In fact, in these examples many discoidal structures may correspond to sedimentary structures, such as tool marks, overload structures, wrinkle marks, pseudofossils and even ring-shaped microbial colonies^8^.

### Geologic setting

**Figure 1:**
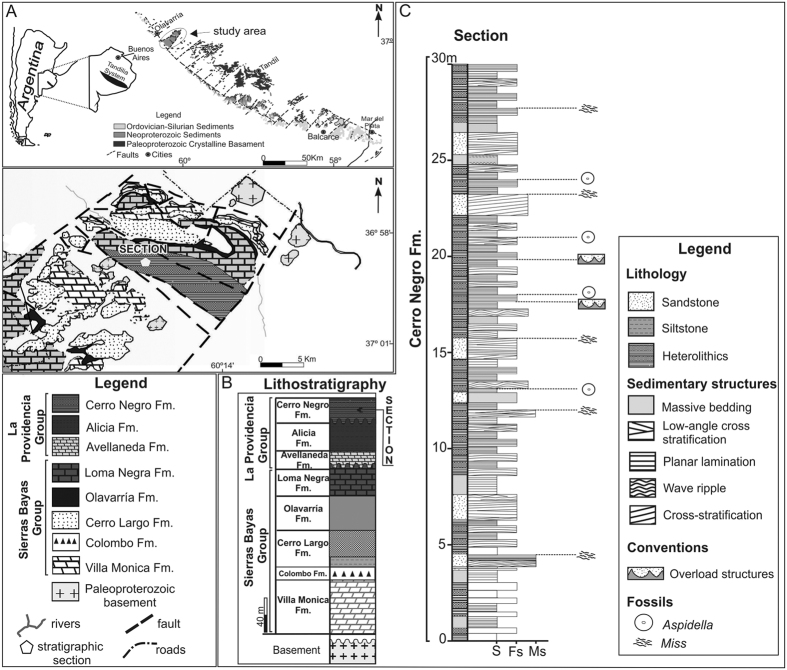
(**A**) Location of the Tandilia System, Buenos Aires Province, Argentina, and geologic map of the Olavarría area (map made by MJ Arrouy in Corel Draw X7 software). (**B**) Lithostratigraphic section of Sierras Bayas and La Providencia Groups. (**C**) Stratigraphic section of the shallow marine and tidally influenced facies of the Cerro Negro Formation. Fm.–Formation, S–Siltstone, Fs–Fine sand, Mf–Medium sand, MISS-Microbially Induced Sedimentary Structure.

The Tandilia System is a 350 km long northwest-to-southeast orographic belt located in the southern of the Buenos Aires Province (Fig. 1A). The unit encompasses igneous and metamorphic rocks of the Paleoproterozoic basement covered by Neoproterozoic sedimentary successions. In the Olavarría area, the stratigraphic column shows ~250 m thickness (Fig. 1B) and is composed of the lower Sierras Bayas Group^26^ and the La Providencia Group^27^, which is subdivided in the Avellaneda, Alicia and Cerro Negro Formations. The Sierras Bayas Group is separated from the overlying La Providencia Group by an erosional unconformity related to eustatic sea-level drop. However, the poor age constraint of the unit precludes a precise estimation on the time range of this hiatus. The Cerro Negro Formation exceeds 100 m in thickness and consists of centimeter-to-decimeter tabular and lenticular beds of terrigenous rocks, arranged as cyclic intercalation of massive and trough cross-bedding fine-grained sandstones, massive red mudstones and heterolithic facies (Fig. 1C). This association indicates traction currents/waves alternating with periods of slack water. The base of the unit shows several levels recording microbially induced sedimentary structures (MISS) and rare mud cracks, which suggests that the sedimentation in this part of the succession occurred under shallow water conditions with sporadic subaerial exposure, typical of a deposition in subtidal environment.

The paleontological content of the Neoproterozoic units of the Tandilla System is poorly known. The supposed presence of *Cloudina*^28^ is based on specimens recorded in thin sections of micritic limestones of the Loma Negra Formation. However, because those remains are poorly preserved, their biogenic origin is controversial, and additional specimens are necessary to confirm or refute their taxonomic identification. Acritarchs described in the Cerro Largo and Cerro Negro formations consist of simple sphaeromorphs, such as *Synsphaeridium* sp., *Trachysphaeridium* sp. and *Leiosphaeridia* sp., compatible with an Ediacaran age for both units^5^.

### Specimens from the Cerro Negro Formation (La Providencia Group)

**Figure 2:**
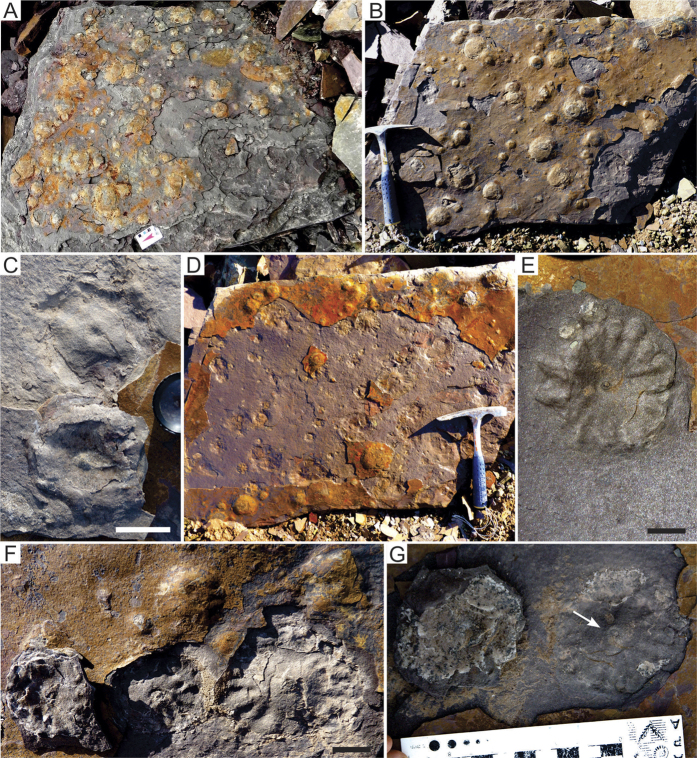
Size variation and morphology of *Aspidella* plexus from the Cerro Negro Formation, La Providencia Group. (**A**,**B**) Samples showing several specimens with great variation in size. (**C**) Medium sized (around 35 mm) discoidal convex specimen with a small boss in the center and very low creases, preserved as negative epirelief (upper) and full relief (lower). (**D**) Pavement showing several specimens with puckered features. (**E**) Detail of negative epirelief specimen with radial folds (puckered features) and invaginated center. (**F**,**G**) Specimens with invaginated centers preserved in negative epirelief, with a full relief specimen counterpart. Scales: (**A**), 10 cm, (**C**,**E**,**F**), 1 cm, (**B**) the hammer is 27,9 cm long.

The newly recorded assemblage from the Cerro Negro Formation includes macroscopic discoidal forms, rare ichnofossils and sedimentary structures ascribed to MISS structures^29^,^30^. The discoidal forms are preserved in at least four stratigraphic levels within a 15 m thick interval at the middle portion of the Cerro Negro Formation (Fig. 1C). The discs occur as dozens to more than a hundred (Fig. 2A,B) forming discrete pavements in the underside of tabular fine-grained sandstone beds interbedded with micaceous red siltstones and mudstones. Flute marks and linear scours (Fig. 2C) occurring in the base of sandstone layers suggest deposition by episodic flows (as tempestites under shallow water conditions).

The discoid- to ovoid-shaped forms occur as low positive epirelief of distinct sizes and show an apparent convex surface with a concentric depression surrounding a central rounded projection (Fig. 2A,B). Some specimens, especially the large ones, are strongly ornamented with radial grooves (Fig. 2E). The convex specimens are isolated individuals or are grouped in small localized clusters (Fig. 2A,B). The individuals have diameters ranging from 6 mm to 140 mm, with the majority of the specimens reaching 10 mm to 26 mm. The smallest individuals (>30 mm in diameter) are the most frequent. They are conspicuously rounded and completely smooth (Fig. 2A,B). The specimens ranging from 30 mm to 65 mm have a small boss in the center reaching maximum height of 3 mm (Fig. 2C). When detached from the fine sandstone bed, each specimen comprising full relief shows a somewhat lenticular transverse section with a lower surface bearing irregular radial folds and grooves and a central rounded pit, leaving its corresponding impression as negative epirelief (Fig. 2D–G). They commonly show an invaginated center, creases and strong radial grooves extending from the center to margin of the disc (Fig. 2E). The medium-sized discs are enclosed by a single circular ridge and preserve typical puckered features in their counterparts (Fig. 2F,G). The largest discs comprise convex forms (positive epirelief) with diameters varying from 65 mm to 100 mm. Their morphology resembles that of the medium-sized discoidal forms.

**Figure 3:**
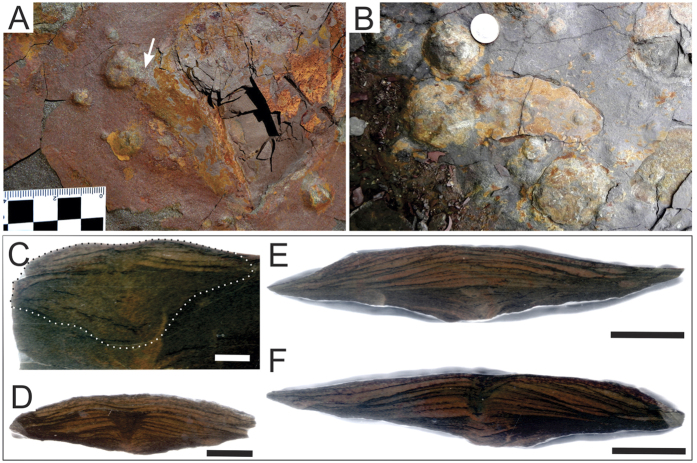
Macro and microscopic diagnostic features of Argentinian discoidal fossils. (**A**) Positive epirelief view showing a structure similar to an attached stalk and frond. A white arrow points the putative connection between the holdfast and the stem. (**B**) Detail of a complete specimen, including a structure interpreted as a possible set of holdfast and frond. Note that the structure that supposedly corresponds to a recumbent frond overlaps at least four small discs. (**C**–**F**) Two full-relieves of discs in cross section ascribed to *Aspidella* (**C**–**F**) are parts and counterparts of the section). Scales: (**B**), the coin has 2.5 cm in diameter; (**C**–**F**), 1 cm.

Apart of the abundant discs, some extremely rare forms (<1% of the analyzed specimens, Fig. 3A,B) show a somewhat straight structure close to the central part of the disc that resembles frond-like structures. These putative fronds are preserved in positive epirelief as attached to their corresponding holdfasts by a single stem that emerges from the central portion of the discs (Fig. 3A). The slightly curved frond-like structures range from 50 mm to 70 mm and apparently have a delicate and thin central stem that divide symmetrically the supposed petal. The possible petalodium emerges few millimeters above the frond-like structure and holdfast junction (Fig. 3B) and resembles the charnid morphology^31^. However, the absence of any visible ornamentation (as internal features and rays) makes it impossible to assign the few available specimens with one specific rangeomorph taxa.

Thin perpendicular sections show various preservational features that are similar to those also observed in the material from the Fermeuse Formation^9^ (Fig. 3C–F). These include a prominent “V” shaped invagination at the central portion of the disc that in some cases deform the underlying laminae (Fig. 3F), as well as slumping and complex filling by sand (Fig. 3C–F). Some specimens (Fig. 3D–F) show a particular convex-up laminated sandy filling pattern.

**Figure 4:**
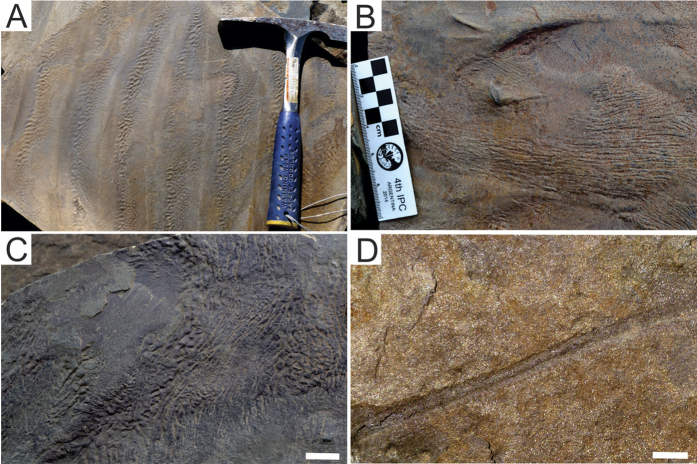
Wrinkle structures of the Cerro Negro Formation, La Providencia Group. (**A**) Several small and simple traces on a wave rippled surface. (**B**) Low relief *Arumberia* type structure with aligned crests developed on the upper surface of a ripple marks. (**C**) Elephant skin structure showing typical reticulate and wrinkly pattern. (**D**) Detail of bed-parallel bilobed trace fossil assigned to cf. *Archaeonassa*. Scales: (**A**), the hammer is 27,9 cm long (**C**), 2 cm; (**D**), 1 cm.

The MISS structures developed on fine-grained sandstone substrates are common in the intermediate portion of the Cerro Negro Formation (Fig. 4). Basically, the biogenicity of those structures can be attested by (a) their occurrence in depositional facies indicating clear water, moderate wave energy and quartz sand bottoms^32^, (b) variable morphologies reflecting local hydrodynamic conditions^32^, and (c) particular textures characterized by crinkly carbonaceous laminae with trapped clastic grains^33^. The presence of elongate and bifurcated forms with flat-topped crests separated by parallel shallow depressions (Fig. A), which rarely form honey-comb configuration suggests that these can be ascribed to *Kinneyia* wrinkle structures. The structure show in Fig. 4B is characterized by slightly curved subparallel flat-topped ridges (locally bifurcated) with height less than 0.2 mm and separated by linear grooves. The morphological complexity of these forms allows us to associate them with the problematic fossil *Arumberia* (especially the *Arumberia banski*^30^, interpreted as a structure formed by very complex and non-actualistic type of microbial community that colonized very shallow waters^30^). Other wrinkle marks show irregular reticulate pattern formed by coalescent nodules and asymmetrical polygons, which are typical of “elephant skin” structures (Fig. 4C). The recurring MISS associated with the fine-grained sandstone beds are mat deformation structures strongly folded and curved (Fig. 4A). The presence of MISS in fine-grained sandstone beds suggests that the substrates were continuously sheltered by microbial mats, reinforcing the intrinsic association between biomats and the preservation of the *Aspidella* discoidal holdfast^31^. Ediacaran terrigenous sediments typically show low degree of bioturbation, which also contributes to the preservation of the basal protuberance of *Aspidella*^34^. Despite of this, recent studies indicate that those organisms may tolerate moderate levels of organic activity in the substrate^35^.

The ichnofossils are very rare and occasionally occur associated with mat deformation wrinkles. Bilobed structures preserved in positive epirelief are characterized by unbranched and slightly curved horizontal traces with two longitudinal transverse ridges separated by a central depression (Fig. 4D). They usually are found below the MISS and are similar to the ichnogenus *Archaeonassa*. This type of trace fossil was previously interpreted as an undermat tunnel made by a bilaterian organism near the sediment-water interface^36^.

### Affinity of Cerro Negro discoidal fossils

**Figure 5:**
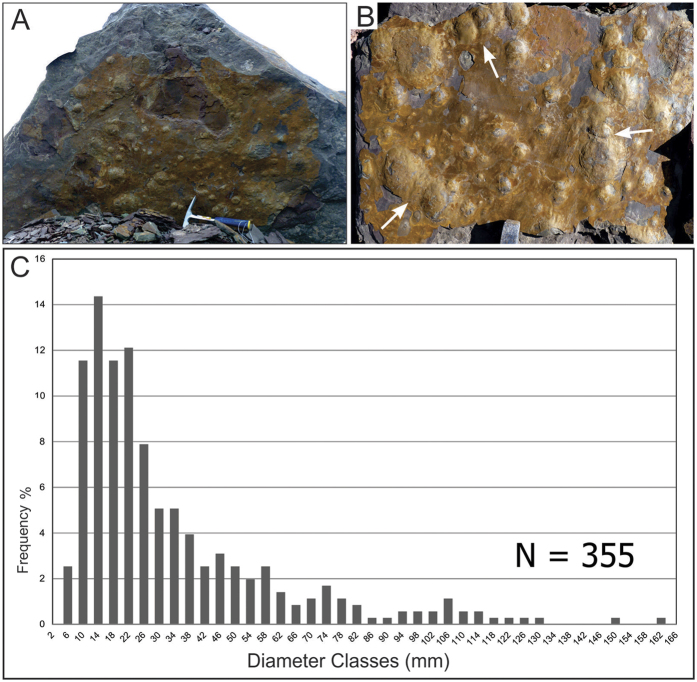
*Aspidella* specimens from the Cerro Negro Formation, La Providencia Group. (**A**) A hundred specimens of discs preserved in a pavement. Note the predominance of specimens smaller than 20 mm and some “giant” specimens with maximum size of 150 mm. (**B**) Pavement showing a large number of discs with different sizes. Note the lack of overlapped specimens and the slightly deformation in the tangent discs indicated by white arrows. (**C**) Size distribution of all *Aspidella* in the pavement sample showed in (**A**). Scale (**A**,**B**) the hammer is 27, 9 mm.

Discoidal structures in sedimentary rocks can be formed by distinct abiotic and biotic processes. Various sedimentary discoidal structures may derive from concretions, molds and casts of nodules, gas and fluid escape conduits, mounds and craters^37^. The stratigraphic interval where the discoidal structures were recorded is mainly characterized by sandstones deposited in shallow water conditions with no evidence of methane or other hydrocarbon seeping. Indeed, the shape (in plan- and cross-section views) and dimensions of discoidal structures are incompatible with those observed in mud volcanos, mounds, conduits, and domes associated to seeping. Not surprisingly, gas and fluid scape microstructures are missing in the polished slabs (Fig. 3). Evidences of concretions or nodules are also absent (Fig. 3). We also exclude other inorganic processes, such as scratch circles made by wave-induced rotation of anchored objects or even that they may represent giant foraminifers^38^,^39^. Hence, based on the evidences below, we think that an exclusively organic origin is the most plausible explanation for the origin of the discoidal structures of the Cerro Negro Formation, mainly due to: (a) lack of tool marks and incomplete, simple, or double rings produced by partial rotation of stalked objects in the same bedding planes where they are abundant (or organisms); (b) radial circles are rare and, when observed, they are not concentric as in the fake *Kullingia* of Newfoundland^38^; (c) absence of micro and thin radiate pattern as found in false discoidal fossils (e.g., *Ediacaria* samples from Russia^8^); (d) the boss or tubercle of each specimen is not perfectly centered, which points to some morphological variation more common of structures of biologic origin (Fig. 2C–G); (e) absence of tube-like or channelized features or conduits in the internal part of the discs linking to the central boss, that could be interpreted as fluid or gas scape structures (Fig. 3C–F); (f) the discs are developed tridimentionally inside and above the substrate level in several size classes (Fig. 5); (g) presence of distinct forms (morphs^9^) represented by hundreds of specimens (Figs 2 and 3) and rare, but diagnostic, frond-like projections; (h) specimens that are closely preserved are deformed only at the touching margins, indicating that they are not coalescent and, thus, each individual is an independent entity (Fig. 5B–i); the lack of overlapped specimens rules out that they could be traces, or even scratch circles (Fig. 5B).

Preservation, general morphology, number of specimens, and their shape and size classes are all similar to that observed in typical *Aspidella* specimens. In particular, the invagination at center, the presence of a central boss (Fig. 2C) as well as the marginal grooves that can form puckered features (Fig. 2D–G) are all structures typically observed in *Aspidella*. Convex-up laminations in some specimens (Fig. 3D–F) can be associated with the collapse of the organism during sandy infilling events, in a typical pattern previously described^9^. In some specimens, the projected central structure could be interpreted as the insertion of a stem-like structure^9^.

The absence of specimens with concentrically ornamented central disc, irregular radial structures and concentric ridges and grooves precludes classifying them as *Ediacaria* or *Spriggia*^2^. Also, the absence of branching radial segments rules out their assignment to *Hiemalora*^2^; similarly, their puckered pattern is very distinctive from the concentrically increasing lobes or tentacle-like delicate spokes of the genus *Mawsonites*^40^, or even other concentric-bearing discoidal fossils^8^,^41^.

It is important to note that, despite several efforts to understand the taxonomy, taphonomy and paleoecology of the early discoidal fossils, no formal taxonomic revision was so far proposed for the *Aspidella* species and other possible synonymous taxa and related forms^2^,^9^,^42^,^43^. Considering this, we include the Cerro Negro specimens as belonging to the *Aspidella* group, or “plexus”^8^.

### Paleoecology and Taphonomy: implications for the Age of the La Providencia Grouph

The large density of individuals (~500 specimens per m^2^) observed in various pavements (Fig. 5A–B) suggests that the Cerro Negro discs lived in high-density populations, as previously observed in other Ediacaran occurrences^9^. This pattern of preservation is commonly found in other worldwide *Aspidella* records and is also noted in modern sessile benthic communities with high juvenile mortality^9^,^43^. The size class distribution of the Cerro Negro specimens (Fig. 5C) also suggests no size-selection prior to the final burial. Yet, they were preserved in sandstones generated by high energy sedimentary processes, indicating that the community was smothered by episodes of rapid sedimentation. The predominance of small individuals indicates that the original living population was mainly composed of minute specimens. The size variation in individuals from the same bedding plane also indicates that the specimens with different dimensions (or even distinct morphologies) are not restricted to a particular bedding plane or strata. Consequently, they are not limited to a specific environment or depositional setting within the examined sedimentary succession. Unlike previously reports^43^ our data suggest some relationship between size distribution and morphology of the discs. For example, the small individuals (<14 mm) are usually smooth, whereas the large ones (up to 70 mm) are puckered. This could denote the preservation of individuals in different ontogenetic stages^9^,^43^, bearing distinct morphological characters.

The preservation of specimens from the Cerro Negro Formation is typical of “death mask” style^1^ reported for *Aspidella* specimens by previous authors^9^ being compatible with the three hyporelief morph types. As commented above, some are also characterized by creases and folds, and may correspond to external molds of the upper surface of holdfasts (i.e., puckered morphology^43^). Various features found in the Cerro Negro assemblagemm are typical of the “Fermeuse-style” of Ediacaran fossil preservation^44^, such as: (a) the density and abundance of disc-shape fossils (*Aspidella*) in various distinct bedding planes at the base of sandstones, representing event beds; (b) the skewed size distribution of the specimens occurring in the same bedding plane with predominance of smaller ones; c) the presence of rare trace fossils; and (d) the rarity of fronds and other rangeomorphs.

However, despite the similarities of both assemblages is noteworthy that the Cerro Negro Formation was deposited in very shallow water conditions (subtidal to tidal setting) whereas the Fermeuse Formation was deposited below fair weather wave base by storm- or turbidite-induced events. In addition, due to the strong association with microbial mats and shallow water settings, the preservation of the probable *Aspidella* from South America also resembles that of the “Flinders-style”^43^. The three dimensional preservation of the studied specimens, including both flat to convex forms^9^ as well as the puckered ones^2^,^43^ may represent one of the most complete spectrum of preservation of members of the *Aspidella* group.

The discoidal fossils are more commonly found in Ediacaran deposits, but there are also scattered occurrences in Cryogenian^2^ to Early Paleozoic successions^45^. However, as commented above, diagnostic features indicative of older (Cryogenian) or younger organisms (Early Cambrian, as *Nimbia* or *Tirasiana*^46^) are lacking, which reinforces the assignment of the Argentinian discs to Ediacaran *Aspidella* group^9^. Despite some uncertainties about the precise age of the studied Neoproterozoic discoidal fossils^2^,^42^, the occurrence of *Aspidella* constrains the age of the La Providencia Group to the terminal Ediacaran^5^,^9^. Additionally, the presence of ichnofossils in the same assemblage reinforces an age no older than 565 Ma^47^.

### Paleobiogeographic distribution: implication for Gondwana reconstruction

**Figure 6:**
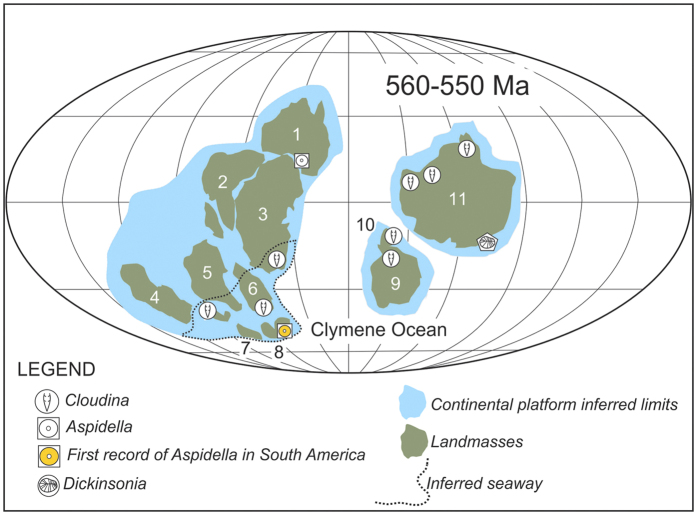
Schematic reconstruction of the Southeastern Gondwana paleogeography during the end of Ediacaran with fossil occurrences. 1-Australia, 2- India, 3-Antarctica, 4-West Africa, 5-Congo–São Francisco, 6- Kalahari, 7-Paraná, 8-Río de la Plata, 9-Amazônia, 10-Río Apa, 11-Laurentia (map made by L.V. Warren in Corel Draw X7 software).

The Argentinian geotectonic province of Tandilia was located in the southwestern portion of the Río de La Plata Craton and corresponds to a narrow strip composed of Paleoproterozoic basement units covered by a slightly deformed Neoproterozoic succession. Available paleomagnetic reconstructions for the Río de La Plata Craton during the Upper Ediacaran (575 Ma) indicate that this plate was separated from Laurentia and probably it was linked with the São Francisco craton in intermediate to low latitudes^48^. It is probable that at 550 Ma these cratonic masses were already part of the proto-Gondwana supercontinent being isolated from the Amazonia/Río Apa microcontinents by the short-lived Clymene Ocean^49^ (Fig. 6). During the Upper Ediacaran, the paleogeographic scenario of the proto-Gondwana indicates a prominent open passive margin to the east^49^ in which several carbonate platforms and shallow marine successions were deposited. The position of the Río de La Plata^48^,^50^ in the context of the proto-Gondwana reinforces the hypothesis of oceanic opening to the east and deposition of the upper portion of the La Providencia Group under fully marine conditions (Fig. 6). Evidences of marine deposition in the upper portion of the La Providencia Group is attested by the presence of acritarchs, tidally influenciated sedimentary facies^27^ and the Ediacaran discoidal fossils described here.

Recent reconstructions support a marine ingression over continental areas in the eastern proto-Gondwana^18^, suggesting that the carbonate platforms represented by the Bambuí (Brazil), Nama (Namibia) and Arroyo del Soldado Groups (Uruguay) as well as the Taylor Formation (Antarctica) were developed in the same shallow eperiric sea. In this context, the marine deposition of the Cerro Negro Formation extends the scenario of widespread tidal flats opened to the east to as early at the end of Ediacaran. The complete closure of the Clymene Ocean along its 3000 km length took place in the early to mid-Cambrian^51^. This geotectonic event was responsible for the deformation of the short-lived basins in the paleo margins of the proto-Gondwana, encompassing sedimentary successions in Africa and South America.

The presence of Ediacaran fossil assemblages in Brazil, Namibia, Antarctica and now in Argentina reinforces the hypothesis of a vast seaway that connected to the Clymene Ocean during the terminal Ediacaran^18^,^52^,^53^. Despite its indisputable paleogeographic significance, the Cerro Negro biota is the first record of Ediacaran soft-bodied macrofossils in South America. This opens new avenue to our understating on the composition and ecology of the Ediacaran life in the shallow water settings developed in the first marine basins of the Gondwana paleocontinent.

## Methods

We collected specimens from a 15 m thick interval of tabular, fine-grained sandstones in the Cerro Negro Formation. Numerous meter-sized slabs were extracted from outcrops and quarry walls. In addition, samples of fallen rock were also collected in the mining area of the Cementos Avellaneda S.A, Olavarría, Argentina. In laboratory, samples were prepared according standard paleontological techniques, and then specimens were measured with digital calipers. Slabs and specimens were photographed and analyzed regarding morphology and taphonomy, including determinations of size classes, thicknesses, modes of preservation (epirelief, hyporelief, full relief), presence of coalescing specimens and presence (or absence) of radial or concentric ornamentations. Thin sections, perpendicularly cutting the discoidal structures, were also prepared, analyzed and imaged. The studied specimens are deposited in the Centro de Investigaciones Geológicas – CONICET – Universidad Nacional de La Plata, La Plata, Argentina.

## Additional Information

**How to cite this article**: Arrouy, M. J. *et al*. Ediacaran discs from South America: probable soft-bodied macrofossils unlock the paleogeography of the Clymene Ocean. *Sci. Rep.* **6**, 30590; doi: 10.1038/srep30590 (2016).
